# Predictive value of molecular subtypes and *APOBEC3G* for adjuvant chemotherapy in urothelial bladder cancer

**DOI:** 10.1002/cam4.5324

**Published:** 2022-10-07

**Authors:** Csilla Olah, Henning Reis, Michèle J. Hoffmann, Fabian Mairinger, Saskia Ting, Boris Hadaschik, Ulrich Krafft, Viktor Grünwald, Peter Nyirady, Melinda Varadi, Balázs Győrffy, Andras Kiss, Eszter Szekely, Gottfrid Sjödahl, Tibor Szarvas

**Affiliations:** ^1^ Department of Urology University of Duisburg‐Essen Essen Germany; ^2^ Dr. Senckenberg Institute of Pathology, University Hospital Frankfurt, Goethe University Frankfurt Frankfurt am Main Germany; ^3^ Department of Urology Medical Faculty and University Hospital Düsseldorf, Heinrich‐Heine‐University Düsseldorf Düsseldorf Germany; ^4^ Institute of Pathology, University Medicine Essen, University of Duisburg‐Essen Essen Germany; ^5^ Department of Medical Oncology University of Duisburg‐Essen Essen Germany; ^6^ Department of Urology Semmelweis University Budapest Hungary; ^7^ Research Centre for Natural Sciences, Cancer Biomarker Research Group Institute of Enzymology Budapest Hungary; ^8^ 2^nd^ Department of Pediatrics and Department of Bioinformatics Semmelweis University Budapest Hungary; ^9^ 2^nd^ Department of Pathology Semmelweis University Budapest Hungary; ^10^ Department of Translational Medicine Lund University Lund Sweden

**Keywords:** *APOBEC3G*, bladder cancer, cisplatin, molecular subtype classification

## Abstract

**Objective:**

Although targeted approaches have become available in second‐ and third‐line settings, platinum‐based chemotherapy remains the standard first‐line treatment for advanced muscle‐invasive bladder cancer (MIBC). Therefore, the prediction of platinum resistance is of utmost clinical importance.

**Methods:**

In this study, we established a routine compatible method for the molecular classification of MIBC samples according to various classification systems and applied this method to evaluate the impact of subtypes on survival after adjuvant chemotherapy. This retrospective study included 191 patients with advanced MIBC (pT≥3 or pN+) who underwent radical cystectomy, with or without adjuvant chemotherapy. A 48‐gene panel and classifier rule set were established to determine molecular subtypes according to TCGA, MDA, LundTax, and Consensus classifications. Additionally, 12 single platinum‐predictive candidate genes were assessed. The results were correlated with patients' clinicopathological and follow‐up data and were validated using independent data sets.

**Results:**

Our final evaluation of 159 patients demonstrated better survival in the luminal groups for those who received chemotherapy compared with those who did not. In contrast, no such differences were observed in basal subtypes. The use of chemotherapy was associated with better survival in patients with high *APOBEC3G* expression (*p* < 0.002). This association was confirmed using an independent data set of patients who received neoadjuvant platinum therapy.

**Conclusions:**

The proposed method robustly replicates the most commonly used transcriptome‐based subtype classifications from paraffin‐embedded tissue samples. The luminal, but not basal, molecular subtypes had the greatest benefit from adjuvant platinum therapy. We identified and validated *APOBEC3G* as a novel predictive marker for platinum‐treated patients.

## Graphical abstract

Transcriptome‐based molecular subtyping is an emerging tool for therapy prediction of muscle‐invasive bladder cancer. However, methodological barriers represent a significant obstacle to its routine use. We present a gene panel‐based, simple, low‐cost method that is applicable to paraffin‐embedded tissue samples and can recapitulate the most important subtype classifications. Our results revealed that the luminal but not basal subtype benefited from adjuvant platinum‐therapy.

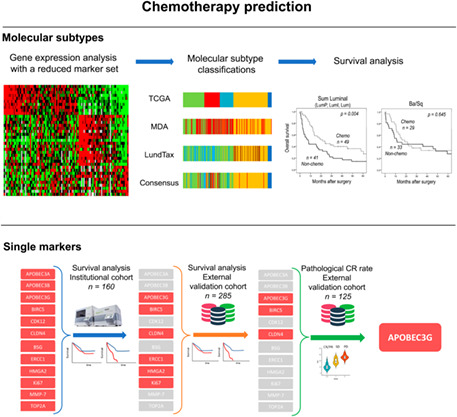

**Abbreviations**
AC: adjuvant chemotherapy
BC: bladder cancer
MDA: MD Anderson
MIBC: muscle‐invasive bladder cancer
L+: lymphovascular invasion
LN: lymph node metastasis
NAC: neoadjuvant chemotherapy
R+: positive surgical margin
RC: radical cystectomy
TCGA: The Cancer Genome Atlas
V+: vascular invasion

**Novelty and Impact**

Transcriptome‐based molecular subtyping is an emerging tool for therapy prediction of muscle‐invasive bladder cancer. However, methodological barriers represent a significant obstacle to its routine use. We present a gene panel‐based, simple, low‐cost method that is applicable to paraffin‐embedded tissue samples and can reproduce various subtype classifications. Our results revealed that the luminal but not basal subtype benefited from adjuvant platinum therapy. Additionally, we identified and validated *APOBEC3G* as an independent predictor of platinum treatment.

## 1 INTRODUCTION

The standard treatment for muscle‐invasive urothelial bladder cancer (MIBC) is radical cystectomy (RC), which provides a 5‐year survival rate of only 50%.^1^ Therefore, perioperative platinum‐based combination chemotherapy is considered a standard of care (SOC) that improves patient prognosis. Application of preoperative neoadjuvant chemotherapy (NAC) for T2‐T4 and cN0M0 tumors results in 20%–40% pathological complete response rates and is therefore recommended by current guidelines.^2^, ^3^, ^4^ However, upfront RC is frequently preferred because the absolute survival benefit provided by NAC is only 5%–10% at 5 years, and delayed cystectomy may reduce the prognosis in chemotherapy‐resistant patients.^5^ Adjuvant chemotherapy (AC) is recommended for patients with locally advanced (pT3‐4) and/or lymph node‐positive (N+) disease who previously did not receive NAC.^3^ In the past few years, immune checkpoint inhibitor (ICI) therapies have become a new SOC as adjuvant treatment in patients with PD‐L1+ MIBC or as part of palliative treatment in patients who are ineligible or resistant to cisplatin, and more recently, as maintenance therapy following response to platinum treatment.^6^, ^7^ Despite the development of the therapeutic landscape, platinum‐based chemotherapy remains the most frequently used first‐line systemic treatment for urothelial bladder cancer. However, novel agents targeting nectin‐4, TROP2, or FGFR are in clinical development and enrich the second‐ and third‐line therapeutic options.^4^ The spectrum of the therapeutic landscape renders the selection of the most appropriate therapy, and the prediction of platinum sensitivity became of great clinical utility.

Gene expression‐based molecular subtype classifications with 3–6 molecular subtypes have been established based on whole‐transcriptome analyses. Different subtypes have been suggested to have divergent prognoses and responses to platinum and immune checkpoint inhibitor therapies, suggesting that molecular subtype classifications may support therapeutic decision‐making.^8^, ^9^, ^10^, ^11^, ^12^, ^13^ Despite the importance of this field, only a few studies have been published to date, which might be explained by the relatively low number of cases in single centers as well as by labor‐intensive and higher‐cost analytical methods. The few large retrospective studies on the association between gene expression‐based molecular subtypes and response to platinum therapy have provided discordant results.^9^, ^14^, ^15^, ^16^ However, these studies are hardly comparable, as they used partly different classification systems and various treatment settings with different endpoints (pathological response rate, overall, and progression‐free survival). Additionally, none of these studies addressed the adjuvant setting for platinum therapy. We recently established a panel‐based gene expression classifier method, which is able to reproduce various classification systems with an accuracy of 70%–80%, and tested this method on snap‐frozen MIBC samples by using RT‐qPCR.^17^

In the present study, we further optimized the gene panel and applied the analysis to the NanoString platform, which enabled us to extend the method to archival formalin‐fixed and paraffin‐embedded (FFPE) tumor samples. To compare the prognosis between platinum‐treated and untreated patients in each molecular subtype, the method has been applied to a retrospective multicenter cystectomy cohort of MIBC patients with pT3/4 and/or N+ who did or did not receive adjuvant platinum‐based chemotherapy. In addition, we assessed the expression of 12 single genes with a suggested involvement in platinum resistance.

## 2 MATERIALS AND METHODS

### 2.1 Patient cohorts

This study included 191 tumor samples from patients with MIBC who underwent RC at the Department of Urology, University of Duisburg‐Essen or Semmelweis University, Budapest, between 2005 and 2018 (institutional cohort). Inclusion criteria were pT≥3 and/or lymph node positivity (N+) at cystectomy, ≥50% tumor cell content in the available tumor tissue, and no preoperative (neoadjuvant) platinum therapy. Ninety‐five patients received adjuvant platinum‐based chemotherapy (chemo cohort) within 90 days after RC, whereas 96 patients did not receive postoperative chemotherapy (nonchemo cohort). We used overall survival (OS) as the primary endpoint, which was calculated as the time between RC and death or the last follow‐up (last update: July 2021). The study was approved by the institutional ethics committees (15‐6400‐BO and TUKEB 55/2014) and performed in accordance with the Declaration of Helsinki. For external validation, two previously published patient cohorts were used: one treated with NAC for MIBC (GSE169455, *n* = 124) and another with MIBC patients who underwent RC treatment without perioperative chemotherapy (GSE83586, *n* = 161).^16^, ^18^

### 2.2 RNA extraction and gene expression analysis

RNA was isolated from FFPE RC specimens using the RNeasy DSP FFPE Kit (Qiagen, Hilden, Germany), according to the manufacturer's protocol. To minimize contamination by nonmalignant tissues, macrodissection was performed, and only previously marked tumor areas with >50% tumor cell content were used for RNA extraction. RNA concentrations were measured using a Multiskan GO microplate spectrophotometer (Thermo Scientific, Waltham, MA, USA). The inclusion criteria for NanoString analysis were an RNA concentration of >20 ng/μl and OD 260/280 ratio between 1.7 and 2.3, and 260/230 ratio between 1.8 and 2.3. NanoString analysis with a custom gene panel of 48 subtype‐specific and 12 additional single genes (Table S1) were performed on the NanoString nCounter Analysis System (NanoString Technologies, Seattle, WA, USA). For data analysis, nSolver software (version 4.0) was applied. Gene expression levels were normalized to the geometric mean of two reference genes (*GAPDH* and *TBP*), six internal positive controls, and eight internal negative controls. Eight of the 191 (4%) samples were excluded due to their low assay efficiency. Further, 23 patients with distant metastasis at RC were excluded from data analysis, leaving 160 patients for further analyses. In addition, one patient in the nonchemo cohort was excluded from the survival analyses due to unavailable survival status. Thus, the final institutional cohort included 79 nonchemo patients and 81 patients who received adjuvant chemotherapy.

### 2.3 Molecular subtype classifiers

In a previous study, we presented a gene panel‐based classifier method for molecular subtype classification according to the mRNA‐based Consensus, LundTax, MDA, and TCGA molecular subtype classifications.^17^ In the present study, we further optimized and condensed our panel and improved the marker sets in order to increase the efficacy of identifying tumors with neuronal subtype,^19^, ^20^ which resulted in an improved set of 48 genes, that is well compatible with the NanoString nCounter platform (for details, see supplementary materials). The marker set covered six tumor cell‐specific (luminal, basal, squamous, neuronal, epithelial‐to‐mesenchymal transition (EMT), and in situ *carcinoma* [CIS]) as well as three stroma‐related gene signatures (p53, extracellular matrix (ECM)/smooth muscle (SM), and immune cell‐specific) (Table S1). Rule sets for each subtype classifier were in silico developed and validated as described in our previous study and in the supplementary material.^17^ Briefly, the classifiers were optimized on published data sets (TCGA [https://tcga‐data.nci.nih.gov/tcga/], MDA [GSE48075], and Lund [GSE83586]) in order to achieve the highest overlap between the original transcriptome‐based classifier and our gene‐panel‐based classifier.

### 2.4 Single markers

In addition to molecular subtype‐specific markers, 12 genes (*APOBEC3A*, *APOBEC3B*, *APOBEC3G*, *BIRC5*, *BSG*, *CDK12*, *CLDN4*, *ERCC1*, *HMGA2*, *MKI67*, *MMP7*, and *TOP2A*) with potential chemotherapy predictive values were added to the gene expression panel (Table S1). The single markers were selected based on literature data^21^, ^22^ as well as our previous or preliminary results.^23^, ^24^

### 2.5 Statistical analysis

Correlations between clinicopathological parameters and molecular subtypes or expression levels of single markers were evaluated using either the chi‐square (for dichotomized variables) or the Wilcoxon rank‐sum test (for continuous variables). Cox univariate and multivariate analyses were performed to evaluate OS. Kaplan–Meier plots were drawn to visualize survival differences. For the 12 single markers, median values were used as cutoff values. Gene expression patterns were visualized using a heatmap (Morpheus, https://software.broadinstitute.org/morpheus/). Statistical analyses were conducted using the SPSS software package (IBM SPSS Statistics for Windows, version 25, IBM Corp., Armonk, N.Y., USA). All tests with a *p* value of ≤0.05 were considered statistically significant.

## 3 RESULTS

### 3.1 In silico testing of our updated marker and rule sets

**FIGURE 1:**
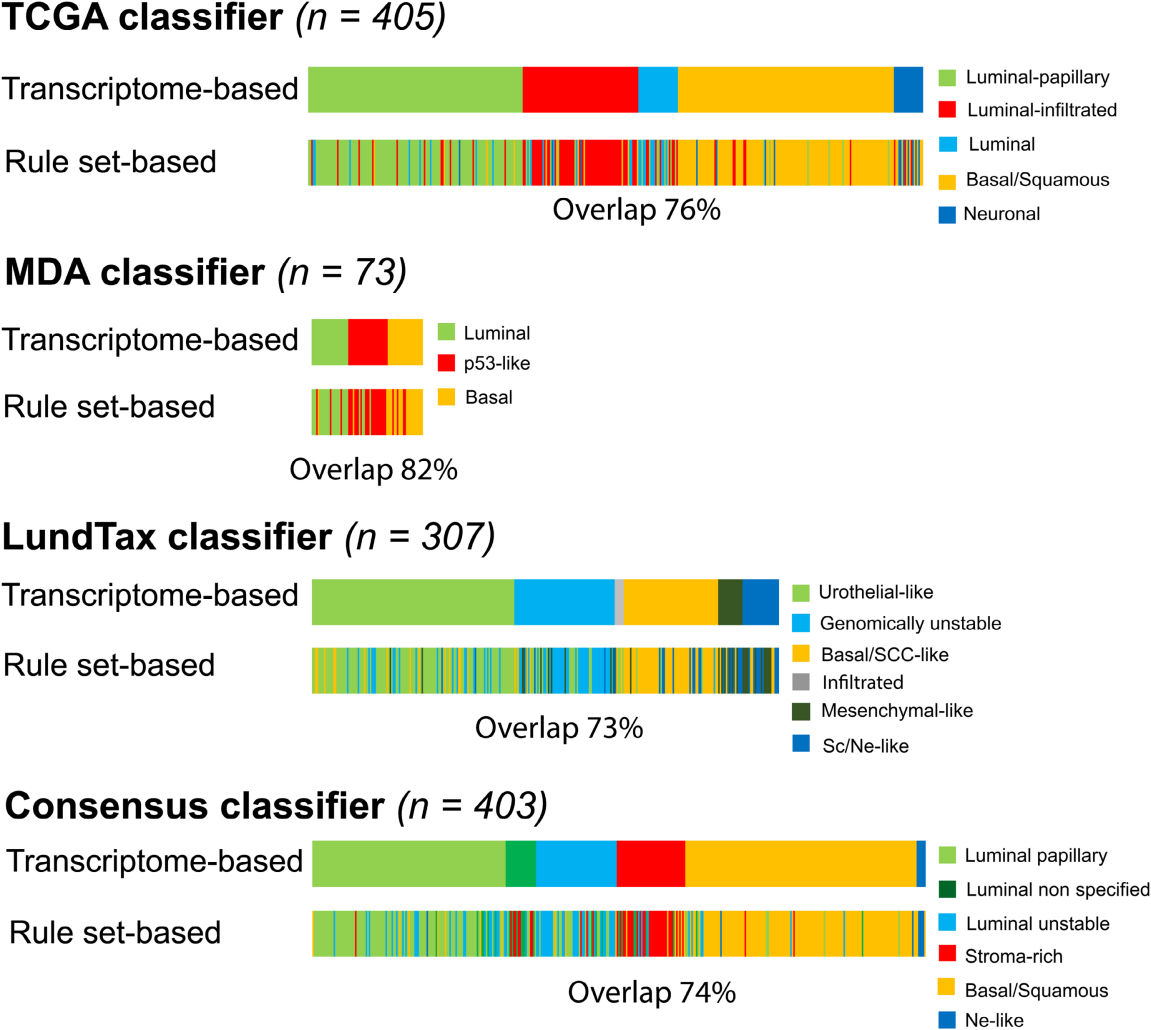
Overlap between the original transcriptome‐based and our 48‐gene rule set‐based classifiers for the TCGA, MDA, LundTax, and Consensus classification systems, as found in published data sets

Classifier rule sets with 48 genes for each classification were in silico elaborated and validated. This revealed a concordance of 76%, 82%, 73%, and 74% between our panel‐based and those of transcriptome‐based classifiers for the TCGA, MDA, LundTax, and Consensus classifications, respectively (Figure 1).

### 3.2 Cohort and follow‐up characteristics

The main characteristics of patient cohorts are presented in Table 1. The two cohorts were comparable in terms of sex, pathological stage (at cystectomy), the occurrence of lymphovascular or vascular invasion, and surgical margin. In contrast, patients' age was lower, and the rate of lymph node metastases was higher in the chemo cohort. In addition, molecular subtype distributions were similar between the chemo and nonchemo cohorts. The median survival of chemo patients tended to be longer compared with nonchemo patients (18.2 vs 8.2 months, *p* = 0.069).

**TABLE 1:** Patients' characteristics

Variables		Nonchemo cohort	Chemo cohort
		*n* (%)	*n* (%)
Total number of patients		79	81
Age at baseline median [range]		72 [48–90]	63 [39–82]
Sex	Male	60 (76)	53 (65)
	Female	19 (24)	28 (35)
Cystectomy data	pT1	‐	1 (1)
	pT2	4 (5)	15 (19)
	pT3	55 (70)	39 (48)
	pT4	20 (25)	21 (26)
	n.a.	0	5 (5)
Lymphovascular invasion	L0	36 (46)	39 (48)
	L+	43 (54)	39 (48)
	n.a.	0	3 (4)
Vascular invasion	V0	63 (80)	56 (69)
	V+	16 (20)	24 (30)
	n.a.	0	1 (1)
Surgical margin	R‐	60 (76)	41 (50)
	R+	18 (23)	16 (20)
	n.a.	1 (1)	24 (30)
Lymph node metastasis at RC	LN0	50 (63)	34 (42)
	LN+	29 (37)	47 (58)
Number of patients died (%)		62 (78)	54 (67)
Follow‐up time in months median (range)		8.2 (0–163)	18.2 (1–157)
Subtype class information			
TCGA	LumP	16 (20)	21 (26)
	LumI	12 (15)	15 (18)
	Lum	14 (18)	13 (16)
	Ba/Sq	33 (32)	29 (36)
	Ne	4 (5)	3 (4)
MDA	Luminal	13 (16)	23 (28)
	Basal	28 (35)	28 (34)
	p53‐like	38 (48)	30 (37)
LundTax	Uro‐like	27 (34)	33 (41)
	GU	14 (18)	13 (16)
	Ba/SCC‐like	24 (30)	23 (28)
	Mes‐like	10 (13)	9 (11)
	Sc/Ne‐like	4 (5)	3 (4)
Consensus	LumP	21 (27)	26 (32)
	LumNS	1 (1)	4 (5)
	LumU	7 (9)	7 (9)
	Stroma‐rich	8 (10)	6 (7)
	Ba/Sq	38 (48)	35 (43)
	Ne‐like	4 (5)	3 (4)

Abbreviations: Ba/Sq, Basal/Squamous; Ba/SCC‐like, Basal/SCC‐like; GU, Genomically unstable; Lum, Luminal; LumI, Luminal‐infiltrated; LumNS, Luminal nonspecified; LumP, Luminal‐papillary; LumU, Luminal unstable; Mes‐like, Mesenchymal‐like; n.a., not available; Ne, Neuronal; Ne‐like, Neuroendocrine‐like; Sc/Ne‐like, Small‐cell/Neuroendocrine‐like; Uro‐like, Urothelial‐like.

### 3.3 Correlations of molecular subtypes with clinicopathological parameters

The results of the following three sections are to be considered exploratory, as no adjustment for multiple testing was performed. Therefore, the findings need to be validated in other cohorts to be generalizable. The basal subtype according to the TCGA, LundTax, and Consensus classification systems was more frequent in women (*p* = 0.018, *p* = 0.021, and *p* = 0.050, respectively). On the other hand, lymphovascular invasion (L+) was less frequent in basal and neuronal (Ne) subtypes compared with the luminal subtypes (TCGA: *p* = 0.018 and *p* = 0.008, MDA: *p* = 0.006, LundTax: *p* = 0.005 and *p* = 0.018, Consensus: *p* = 0.066 and *p* = 0.020). Accordingly, the frequency of lymph node metastasis at cystectomy (LN+) was significantly lower in basal tumors according to each classifier (TCGA: *p* = 0.020, MDA: *p* = 0.008, LundTax: *p* = 0.002, Consensus: *p* = 0.010). The occurrence of vascular invasion was significantly higher in the mesenchymal‐like subtype (LundTax: *p* = 0.014) (Table S2).

### 3.4 Correlations of single markers with clinicopathological parameters

Correlations between patients' clinicopathological characteristics and marker gene expression levels are shown in Table S3. None of the markers was associated with patients' age, L+, LN positivity, or tumor stage. *APOBEC3G* and *MMP7* expression levels were significantly higher in women (*p* = 0.017 and *p* = 0.044, respectively). Lower *MMP7* levels were correlated with vascular invasion (*p* = 0.008), whereas lower *APOBEC3G* and *MKI67* levels were associated with surgical margin positivity (*p* = 0.010 and *p* = 0.018, respectively).

### 3.5 Univariate and multivariate survival analyses of clinicopathological parameters, molecular subtypes, signature scores, and single markers

In the whole cohort, the presence of L+ (*p* < 0.001), positive surgical margin (R+) (*p* = 0.003), the small‐cell/neuroendocrine‐like (Sc/Ne‐like) subtype (LundTax: *p* = 0.021), and low *APOBEC3G* gene expression levels (*p* < 0.001) were associated with significantly shorter OS. According to multivariate analysis, L+ and the Sc/Ne‐like subtype proved to be independent risk factors in the entire cohort (*p* < 0.001 and *p* = 0.010, respectively) (Table S4A, B).

In the nonchemo cohort, the presence of L+ (*p* < 0.001), margin positivity (*p* = 0.013), and LN positivity (*p* = 0.004) were associated with shorter OS, whereas the Ba/Sq subtype (according to the consensus classification) was correlated with improved OS (*p* = 0.049). L+ and margin positivity proved to be independent significant risk factors for OS in the multivariate model (*p* = 0.001 and *p* = 0.012, respectively) (Table S4A, B).

In the chemo cohort, shorter OS was associated with surgical margin positivity (*p* = 0.041), while high *APOBEC3G* expression was significantly correlated with improved OS (*p* < 0.001). According to the multivariate model, in the chemo cohort, high *APOBEC3G* expression tended to be independently associated with superior OS (*p* = 0.057) (Table S4A, B).

We calculated signature scores for each sample according to Table S1 as described earlier and correlated these scores with OS.^17^ Univariate analysis revealed that high neuronal signature scores were associated with shorter OS in the whole and in the nonchemo cohorts (*p* = 0.002 and *p* = 0.004, respectively), while high CIS and immune scores were significantly associated with improved OS in the whole cohort (*p* = 0.020, *p* < 0.001) and both in the nonchemo (*p* = 0.025, *p* = 0.009) and chemo (*p* = 0.049, *p* = 0.040) subgroups (Table S4A). The neuronal and immune signatures remained independent risk factors according to the multivariate models in the whole (*p* < 0.001, *p* = 0.027) and nonchemo cohorts (*p* = 0.05, *p* = 0.050) (Table S4C).

Molecular subtypes (according to TCGA, MDA, LundTax, and Consensus classifications) were not associated with OS in the whole cohort. However, neuronal subtypes showed inferior prognosis according to all subtype classification systems (Consensus, LundTax, TCGA) (Figure S1).

Prognostic values for each of the 12 single‐gene markers in the chemo and nonhemo subgroups are listed in Table S4A. *APOBEC3G* was the only marker found to be associated with OS in the chemo group. Importantly, such a correlation was not observed in the nonchemo group, suggesting a therapy‐dependent prognostic value for this gene (Table S4A).

### 3.6 Comparison of OS between platinum‐treated and untreated patients in molecular subgroups

**FIGURE 2:**
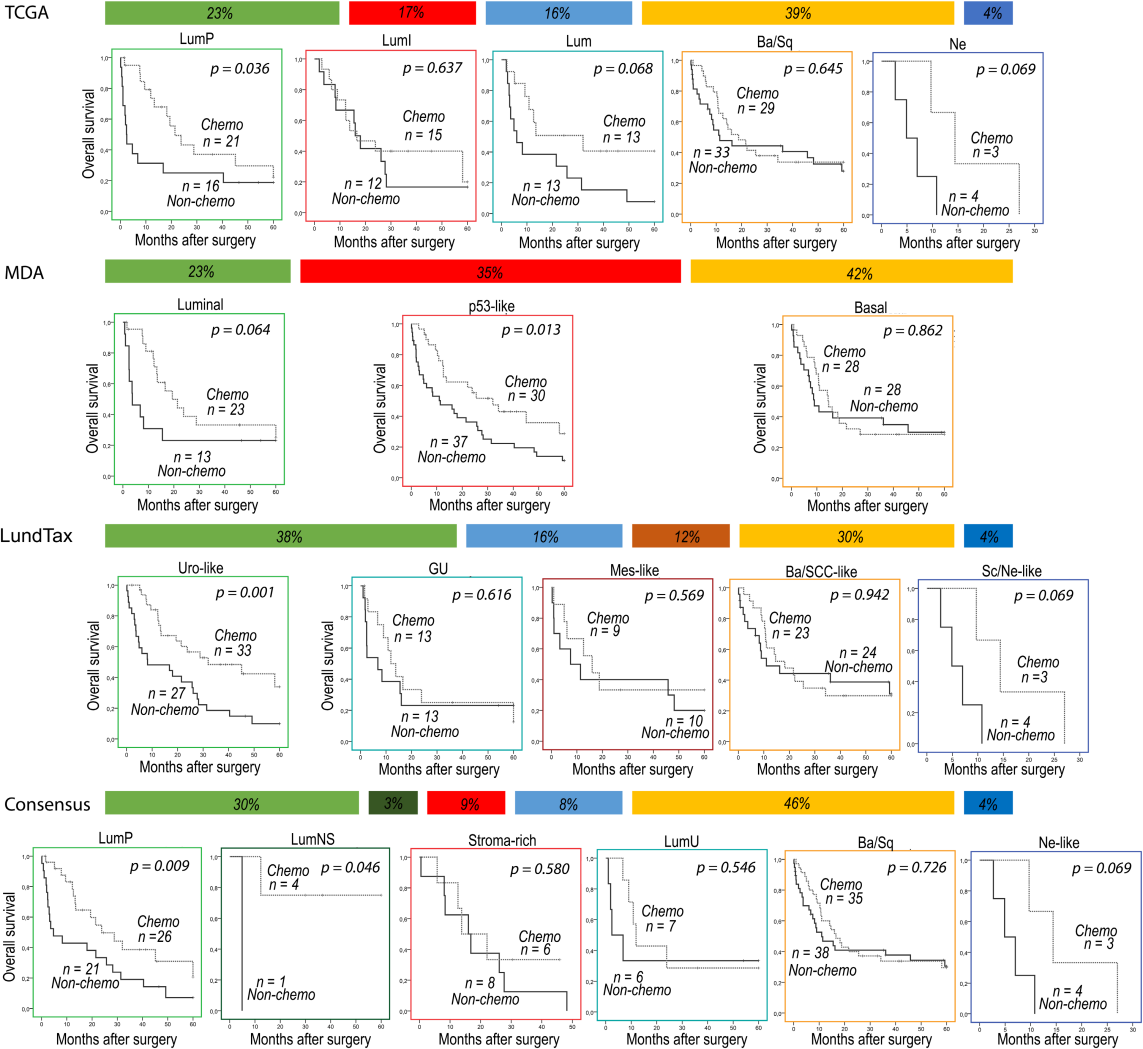
Kaplan–Meier overall survival curves stratified by chemotherapy treatment (chemo vs. nonchemo) in different molecular subtype groups according to TCGA, MDA, LundTax, and Consensus classifications. Ba/Sq, Basal/Squamous; Ba/SCC‐like, Basal/SCC‐like; GU, Genomically unstable; Lum, Luminal; LumI: Luminal‐infiltrated; LumNS, Luminal nonspecified; LumP, Luminal‐papillary; LumU, Luminal unstable; Mes‐like, Mesenchymal‐like; Ne, Neuronal; Ne‐like, Neuroendocrine‐like; Sc/Ne‐like, Small‐cell/Neuroendocrine‐like; Uro‐like, Urothelial‐like

As predictive biomarkers should help to decide whether a patient with a certain molecular pattern will benefit from platinum therapy, a model that first assigns patients into molecular subgroups and then compares survival between those who received and did not receive chemotherapy provides a clinically more appropriate model. Therefore, we performed respective analyses for molecular subtypes and single biomarkers. According to this approach, we found a significantly better OS for the chemotherapy‐treated patients in some of the luminal subtypes, such as luminal papillary (TCGA: *p* = 0.036, Consensus: *p* = 0.009) or urothelial‐like subtypes (LundTax: *p* = 0.001) (Figure 2). In contrast, in basal subtypes, survival rates proved to be similar in the chemo and nonchemo groups (according to all classifications), suggesting that luminal rather than basal subtypes benefit from platinum‐based chemotherapy. Differentiating between various luminal subgroups may be challenging; therefore, we also performed survival analyses for merged luminal (sum luminal) groups according to TCGA, LundTax, and Consensus classifications (Figure S2). Patients with tumors classified into the sum luminal subtype had improved OS in the adjuvant chemotherapy group compared with the radical cystectomy‐only group (TCGA: *p* = 0.004, LundTax: *p* = 0.003, Consensus: *p* = 0.003).

We applied the same approach to the 12 single‐gene markers by dividing cases into marker‐high and marker‐low groups (using the median level as cutoff) and stratifying patients by therapy (chemo vs. nonchemo) (Figure S3). These analyses showed that patients with high *APOBEC3G*, *ERCC1*, and *CLDN4* levels and patients with low *BIRC5*, *HMGA2*, and *MKI67* levels benefited from adjuvant platinum therapy (Figure S3A,B). These results suggest that the above markers may have predictive value for adjuvant platinum therapy. In contrast, no predictive value was found for *APOBEC3A*, *APOBEC3B*, *CDK12*, *BSG*, *MMP7*, and *TOP2A* (Figure S3C, S4).

### 3.7 Validation of the chemotherapy predictive value of single markers on external data sets

**FIGURE 3:**
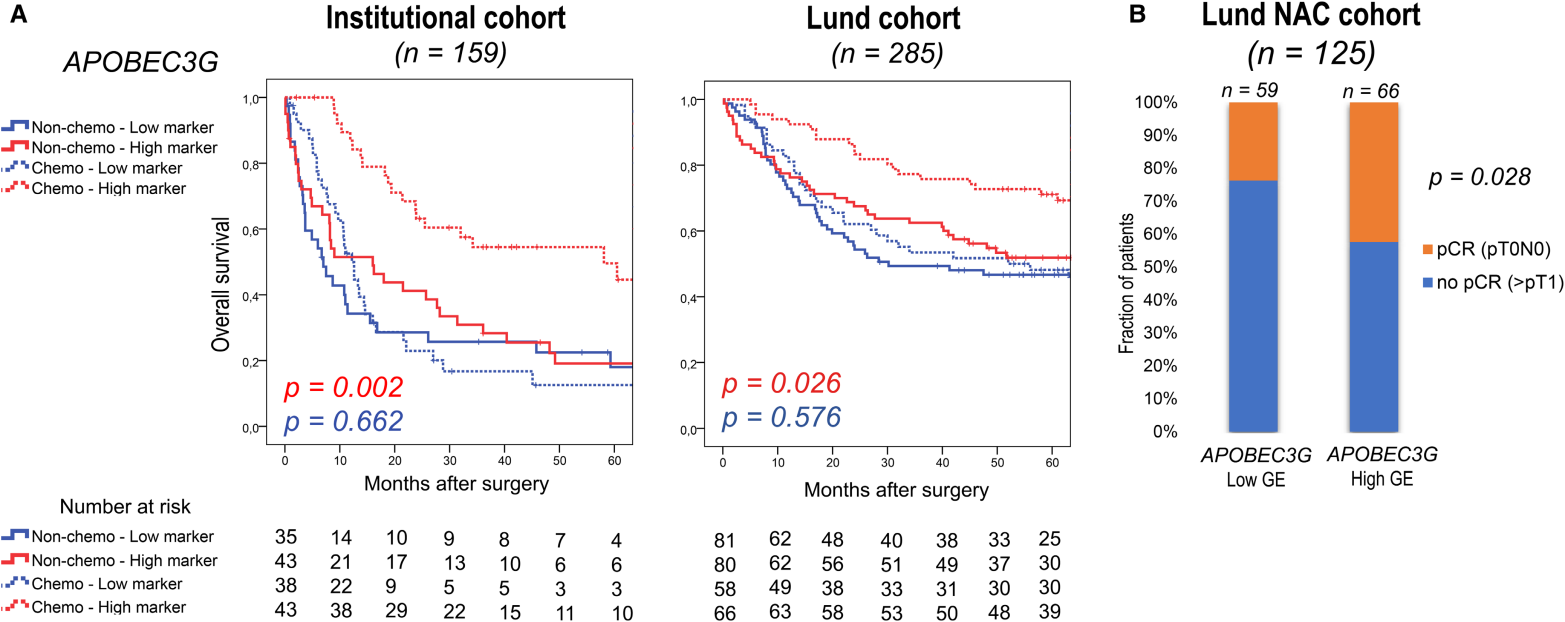
Overall survival stratified by gene expressions of APOBEC3G in our institutional (*n* = 159) and validation (Lund) (*n* = 285) cohorts (A). Pathological response rate for neoadjuvant chemotherapy in the APOBEC3G low and high gene expression groups in the Lund NAC cohort (B). *p* values represent OS difference between platinum‐treated and untreated patients in the subgroups with high gene expression (red) and low gene expression (blue) levels

In order to validate the therapy predictive values of the above‐identified six markers (*APOBEC3G*, *CLDN4*, *ERCC1*, *BIRC5*, *HMGA2*, and *MKI67*) in independent platinum‐treated and untreated cohorts, we used a previously published transcriptome data set of 285 patients who underwent RC with (*n* = 124) or without (*n* = 161) neoadjuvant chemotherapy (Lund cohorts).^16^ We found that patients with high *APOBEC3G* expression had a significantly longer OS in the chemotherapy group (*p* = 0.026), while such a correlation could not be observed in the nonchemo (RC alone) group (*p* = 0.576), confirming the differential prognostic value of *APOBEC3G* expression between platinum‐treated and untreated patients (Figure 3A, Figure S5A). In addition, high *CLDN4* and low *BIRC5* groups were confirmed to have significantly better survival when receiving platinum therapy (*p* = 0.025, *p* = 0.032) (Figure S5C and E). Next, we assessed the pathological complete response (pCR) rates in the high‐ and low‐level groups of *APOBEC3G*, *CLDN4*, and *BIRC5* markers in the Lund NAC cohort. In the *APOBEC3G* high‐expression group, the pCR rate of 42% was significantly higher compared with the 24% found in the *APOBEC3G* low‐expression group (*p* = 0.028) (Figure 3B, Figure S5B). These results, in line with time‐to‐event (OS) analysis, support *APOBEC3G* as a platinum predictive marker in bladder cancer. In contrast, pCR rates were not significantly different between the high‐ and low‐expression groups of *CLDN4* and *BIRC5* (Figure S5D and F).

## 4 DISCUSSION

In the present study, we used a novel panel‐based classifier method to assess the association between molecular subtypes and survival in patients with locally advanced (pT3/4) or lymph node‐positive bladder cancer who did or did not receive adjuvant platinum therapy. Our data show that patients with luminal tumors derive a significant OS benefit from adjuvant platinum chemotherapy, whereas no such benefit was observed in patients with basal tumors. In addition, we identified and validated for the first time *APOBEC3G* as a platinum predictive marker for MIBC.

In recent years, gene expression‐based molecular taxonomy of MIBC has identified discrete subtypes with different prognoses and different sensitivities to systemic treatments. Therefore, molecular subtype classification holds promise for supporting future therapeutic decision‐making. These initial observations are encouraging, but partially discordant; therefore, further validation and prospective testing are needed. Choi et al. reported that the p53‐like molecular subtype shows a poor response to NAC, whereas Seiler et al. demonstrated a survival benefit to NAC in basal tumors.^8^, ^9^ However, these later findings were not supported when considering histopathological response as an endpoint.^9^ A recent study using the same classification (GSC) system in the same therapy setting (NAC) found only partly similar results showing a survival benefit for the so called “nonluminal” subgroup, which was created by lumping the basal, claudin‐low and luminal infiltrated subtypes together. Also here, no association has been reported between molecular subtypes and pathological response.^14^ In a large meta‐analysis of formerly published data sets, a consensus classifier has been defined based on the previously suggested subtype classification systems. This study found no significant survival benefit for any of the subtypes in NAC‐treated patients.^12^ More recently, two independent studies in preoperative (NAC) and salvage chemotherapy settings suggested that patients with basal tumors do not benefit from platinum‐based chemotherapy in terms of survival. Accordingly, both studies could corroborate each other's findings by showing inferior pathological and radiographic responses to platinum‐based chemotherapy in basal tumors. In contrast, luminal tumors proved to have a better cancer‐specific survival and a higher response rate to neoadjuvant or salvage platinum therapy.^15^, ^16^ However, the discrepancies between the findings of the above‐mentioned studies may be explained by the fact that the GSC system classifies different samples into the basal subtype compared to those of the Lund and Consensus classification systems; thus, the GSC‐basal subtypes rather poorly overlap with Lund and Consensus basal tumors.^9^, ^14^, ^16^

In the present study, we assessed the differential survival of patients with distinct molecular subtypes for the first time in the context of adjuvant platinum therapy. For this, we retrospectively selected patients with clinical indications for AC and divided cases into two groups: those who received AC and those who did not. Tumor samples from both patient groups were classified according to various molecular classification systems, and overall survival for each molecular subtype was compared between treated and untreated patients. Our results consequently revealed a significant OS benefit from AC in the luminal subgroups and no benefit in the basal subtypes, independent of the classification system used. However, our results are only in the adjuvant setting and are not directly comparable with any of the other studies but seem to support the notions of the two current studies by Taber and Sjödahl, showing no benefit of platinum therapy in basal tumors.^15^, ^16^

Platinum therapy has been suggested to enhance the antitumor immune response and immunogenic cell death in bladder cancer. Moreover, more abundant immune cell infiltration prior to chemotherapy was significantly associated with an improved response to platinum treatment.^15^, ^25^ Accordingly, Eckstein et al. revealed that cytotoxic T‐cell gene expression signatures are associated with longer OS in MIBC patients who underwent AC.^26^ In line with these findings, our immune gene signature was associated with significantly improved OS in AC‐treated patients but also in the RC (nonchemo) cohort. This raises the question whether the immune gene expression score is predictive or rather prognostic? Our former analyses of an independent cohort of MIBC patients who underwent RC without chemotherapy revealed a more abundant immune signature that was independently associated with favorable OS, which is suggestive of a prognostic association.^17^ Overall, immune cell‐related gene expression appeared to be associated with patients' prognosis in MIBC. However, a more detailed molecular dissection of the immunological microenvironment is warranted in order to identify potentially platinum predictive immune signatures.

A further aim of this study was to test single markers for their potential platinum predictive value. We selected 12 genes from our preliminary study and published literature.^21^, ^22^, ^23^, ^24^ These genes were analyzed in our institutional chemo (AC) and nonchemo (RC only) cohorts, and those markers with suggested predictive values were further tested in an independent dataset of NAC‐ and RC‐treated patients (Lund cohorts). This later validation analysis confirmed the predictive value of three genes, *APOBEC3G*, *CLDN4*, and *BIRC5* for OS. Of the 12 single markers assessed in the present study, only high expression of *APOBEC3G* was associated with significantly higher pathological complete response rates, further confirming the platinum predictive value of this gene.

*APOBEC3G* (apolipoprotein B mRNA‐editing enzyme, catalytic polypeptide‐like 3G) codes a cytidine deaminase and is known to be involved in antiviral defense.^27^ Its expression has been evaluated in various tumors, but no data are available regarding MIBC. To our knowledge, only one study has analyzed *APOBEC3G* expression in urothelial tumor cell lines.^28^ In colorectal cancer, APOBEC3G tissue protein expression was associated with poor prognosis and suggested to be mechanistically involved in the formation of liver metastasis.^29^, ^30^ However, another in vitro study found that high *APOBEC3G* expression reduced the migration ability of human hepatocellular carcinoma cells.^31^ *APOBEC3G* gene and protein expression was significantly more abundant in melanoma compared to adjacent normal tissues, and its higher expression was associated with longer overall and recurrence‐free survival. Moreover, *APOBEC3G* expression is positively correlated with the infiltration of B cells, CD8+ T cells, macrophages, neutrophils, and dendritic cells.^32^ Our present results warrant further analyses regarding the role of *APOBEC3G* in bladder cancer, which may be methodologically hampered due to challenging immunological differentiation between various members of the APOBEC family.^33^

This study is limited by its retrospective nature and small sample size due to the low number of cases in some molecular subtype groups. A further limitation is the availability of only one endpoint of OS in our institutional cohort, as in the adjuvant setting, no radiologically or pathologically measurable lesions are available for monitoring the primary treatment effect. Furthermore, surveillance strategies vary, and real‐world imaging modalities are not as stringently implied compared in clinical trials, rendering disease‐free survival difficult to assess. On the other hand, in the adjuvant setting, patient selection is based on definitive pathological staging, which allows the selection of a more focused patient group. As this is a retrospective study, selection bias between patients who did and did not receive AC may well be present, even if we did not detect differences in subtype distribution between AC treated and untreated patients. The strengths of this study are (1) this is the first study to assess molecular subtypes in the adjuvant platinum setting; (2) the application of the four different classification systems in the same cohort; (3) the use of a new panel‐based, simple, low‐cost method, which is compatible with current clinical routine; and (4) the validation of biomarker results in external data sets.

## 5 CONCLUSIONS

In conclusion, our data suggest that luminal rather than basal tumors benefit from adjuvant platinum‐based chemotherapy. In addition, we identified and validated *APOBEC3G* as a novel predictive marker for first‐line platinum therapy. These observations, when confirmed in prospective studies, may improve therapeutic decision‐making in MIBC.

## AUTHOR CONTRIBUTIONS

**Csilla Olah:** Conceptualization (equal); investigation (equal); methodology (equal); visualization (equal); writing – original draft (equal). **Henning Reis:** Funding acquisition (equal); investigation (equal); supervision (equal); writing – review and editing (equal). **Michèle Janine Hoffmann:** Funding acquisition (equal); supervision (equal); writing – review and editing (equal). **Fabian Mairinger:** Formal analysis (equal). **Saskia Ting:** Investigation (equal); writing – review and editing (equal). **Boris Hadaschik:** Resources (equal); writing – review and editing (equal). **Ulrich Krafft:** Formal analysis (equal); writing – review and editing (equal). **Viktor Grünwald:** Writing – review and editing (equal). **Pèter Nyiràdy:** Resources (equal); writing – review and editing (equal). **Melinda Varadi:** Investigation (equal); writing – review and editing (equal). **Balazs Gyorffy:** Formal analysis (equal); writing – review and editing (equal). **Andras Kiss:** Resources (equal); writing – review and editing (equal). **Eszter Szekely:** Investigation (equal); writing – review and editing (equal). **Gottfrid Sjödahl:** Methodology (equal); writing – review and editing (equal).

## FUNDING INFORMATION

This work was supported by the Wilhelm‐Sander Stiftung (D/106–22012) and IFORES (D/107–137709). T. S. was supported by a János Bolyai Research Scholarship of the Hungarian Academy of Sciences (BO/00451/20/5) and by the New National Excellence Program (ÚNKP‐21‐5‐SE‐3) and the K139059 grant of the Ministry for Innovation and Technology from the source of the National Research Development and Innovation Fund. B.G. was supported by the 2020‐4.1.1.‐TKP2020 grant.

## CONFLICT OF INTEREST

BH reports personal fees and nonfinancial support from AstraZeneca, Amgen, Bayer, BMS, and Janssen; personal fees from ABX, Lightpoint Medical, Inc, and Pfizer; and grant funding from the German Research Foundation.

## ETHICS STATEMENT

The study was performed according to the Declaration of Helsinki and the institutional ethics committee approved the study protocol (15‐6400‐BO and TUKEB 55/2014). Informed consent prior to each treatment was obtained by every patient.

## Supporting information


Appendix S1

## Data Availability

The data that support the findings of this study are available from the corresponding author upon reasonable request.
