# Supplementary material for: Predictive value of molecular subtypes and APOBEC3G for adjuvant chemotherapy in urothelial bladder cancer
Source: Cancer Med. 2022 Oct 7;12(5):5222–32. doi: 10.1002/cam4.5324 (PMC10028049; doi:10.1002/cam4.5324)
Supplement: Supplementary file 1 — Appendix S1 [file CAM4-12-5222-s001.zip › CAM4_5324_Supplementary materials_revised_09.08.docx]

**Supplementary materials**

*Development of a classifier method for molecular subtyping as described earlier*

Our gene panel-based classifier method was developed as described earlier ^1^. As a first step, we selected the genes with the highest discriminating effect between various molecular subtypes according to publicly available datasets and classifiers, such as TCGA, MDA, LundTax, and Consensus classification systems. First, the markers with the highest discriminating effect between different subtypes were determined based on the TCGA dataset ^2^. These genes were further selected prioritizing those, which has been used also in other classification systems (e.g. p53 associated genes / MDA classification or the CDKN2A gene / LundTax) ^3,4^. The finally selected 68 genes covers 6 tumor cell-specific (luminal, basal, squamous, neuronal, epithelial-to-mesenchymal transition (EMT), *in situ carcinoma* (CIS)) and 3 stroma-related gene signatures (p53, extracellular matrix (ECM)/smooth muscle (SM), and immune). Then, we used respective publicly available datasets for the *in silico* development and validation of subtype classification rule sets for the TCGA, MDA, LundTax and Consensus classification systems ^2-5^. For each classification system, two datasets with available transcriptome-based subtype class information were used for the elaboration (training set) and validation (validation set) of our classifier method. From the transcriptome datasets only the selected 68 genes were used for the rule set calculations. Using an automatic cut-off generation each gene expression was scored from 1-5 (resulting in 5 equal 20% percentiles). For each sample, signature scores (basal/squamous, luminal, neuronal, CIS, ECM, EMT, p53-like, immune) were calculated as the mean value of the respective gene expression scores. A stepwise classification of samples was optimized on the training sets by adjusting two parameters; cut-offs for signature score dichotomization (low vs. high for each signature) and the sequence of selection steps into different subtype groups. These two parameters were adjusted until the highest overlap with the original transcriptome-based classification in the training set was reached. The so developed classifier rule sets were applied to the validation sets to evaluate their accuracy in a second cohort. For the definition of the TCGA classifier rule set, we randomly divided the TCGA dataset (https://tcga-data.nci.nih.gov/tcga/) into a training set (n=203) and a validation set (n=202), for the MDA classification we used the GSE48075 dataset (discovery cohort) as the training cohort (n=73) and the TCGA dataset as the validation cohort (n=231), for the LundTax classification, the GSE83586 dataset was divided into a training (n=154) and a validation cohort (n=153), while for the Consensus classification, we divided again the TCGA dataset into a training (n=201) and a validation set (n=202). The above-described method was applied in our previous study ^1^, which has been optimized in the present work as described below.

*Optimization of the formerly developed gene panel and classifier method in this study*

As all signature scores were calculated more than one (4-10) genes, some of them had higher while others lower influence on the final classification, which make possible to reduce the marker set without affecting the accuracy of the method. We observed the lowest accuracy of our method in the identification of the neuronal subtype; therefore, we changed some neuronal genes used for the calculation of the neuronal signature score ^6,7^. Overall, in the present work, we reduced the previously determined marker set from 68 to 48 gene. For this, based on our experiences in our former study we selected genes with lower influence on the subtypes and then excluded these genes for the final classification. These genes were tested *in silico* to confirm their lower specificity for the molecular subtype classification. Supplementary Table 1 summarizing the selected genes used in the present study.

*Supplementary Figures*


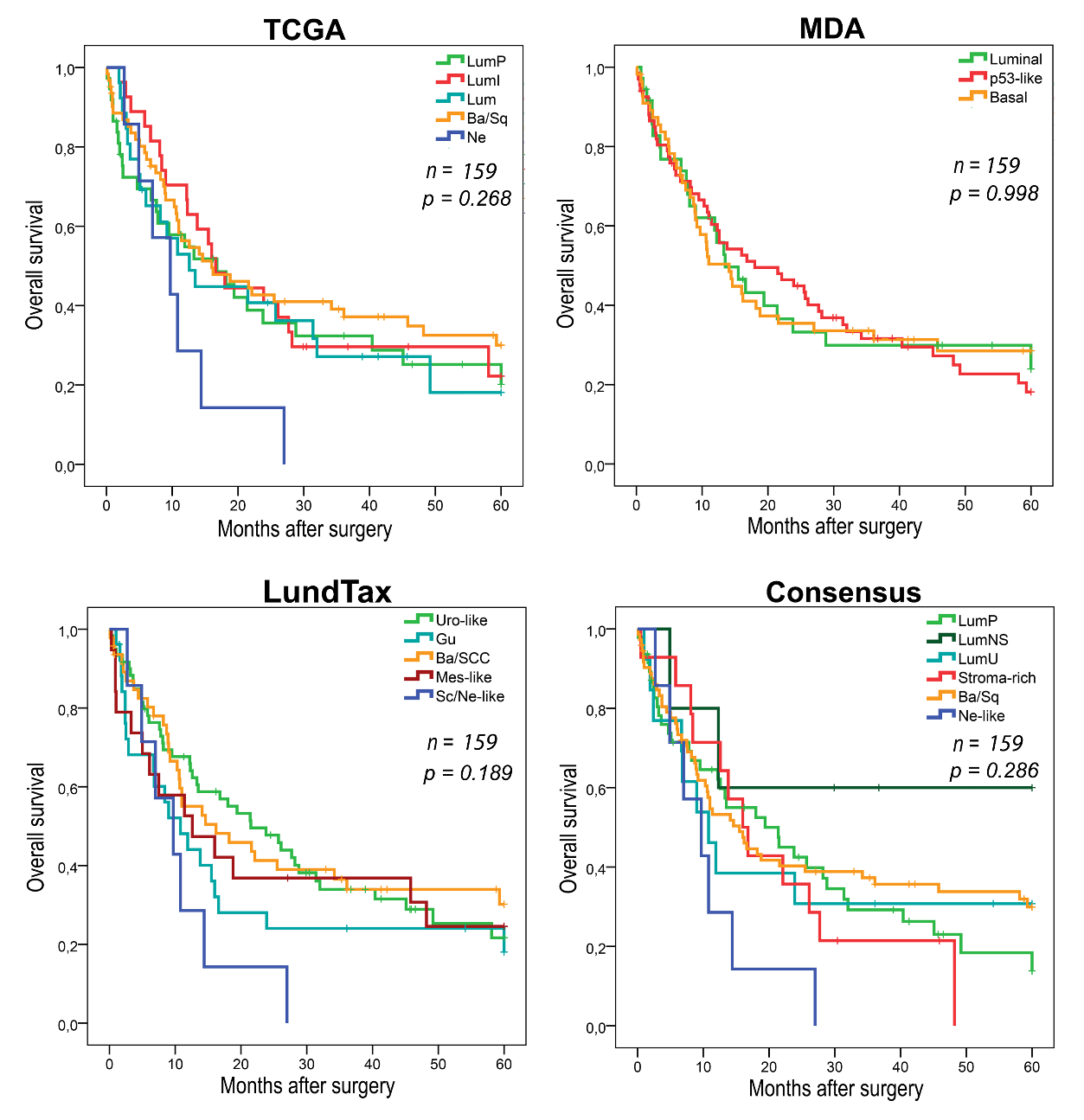


**Supplementary Figure 1.** Kaplan-Meier curves of overall survival stratified by molecular subtypes. LumP: Luminal-papillary, LumI: Luminal-infiltrated, Lum: Luminal, Ba/Sq: Basal/Squamous, Ne: Neuronal, Uro-like: Urothelial-like, GU: Genomically unstable, Mes-like: Mesenchymal-like, Ba/SCC: Basal/SCC-like, Sc/Ne-like: Small-cell/Neuroendocrine-like, LumNS: Luminal non specified, LumU: Luminal unstable, Ne-like: Neuroendocrine-like.


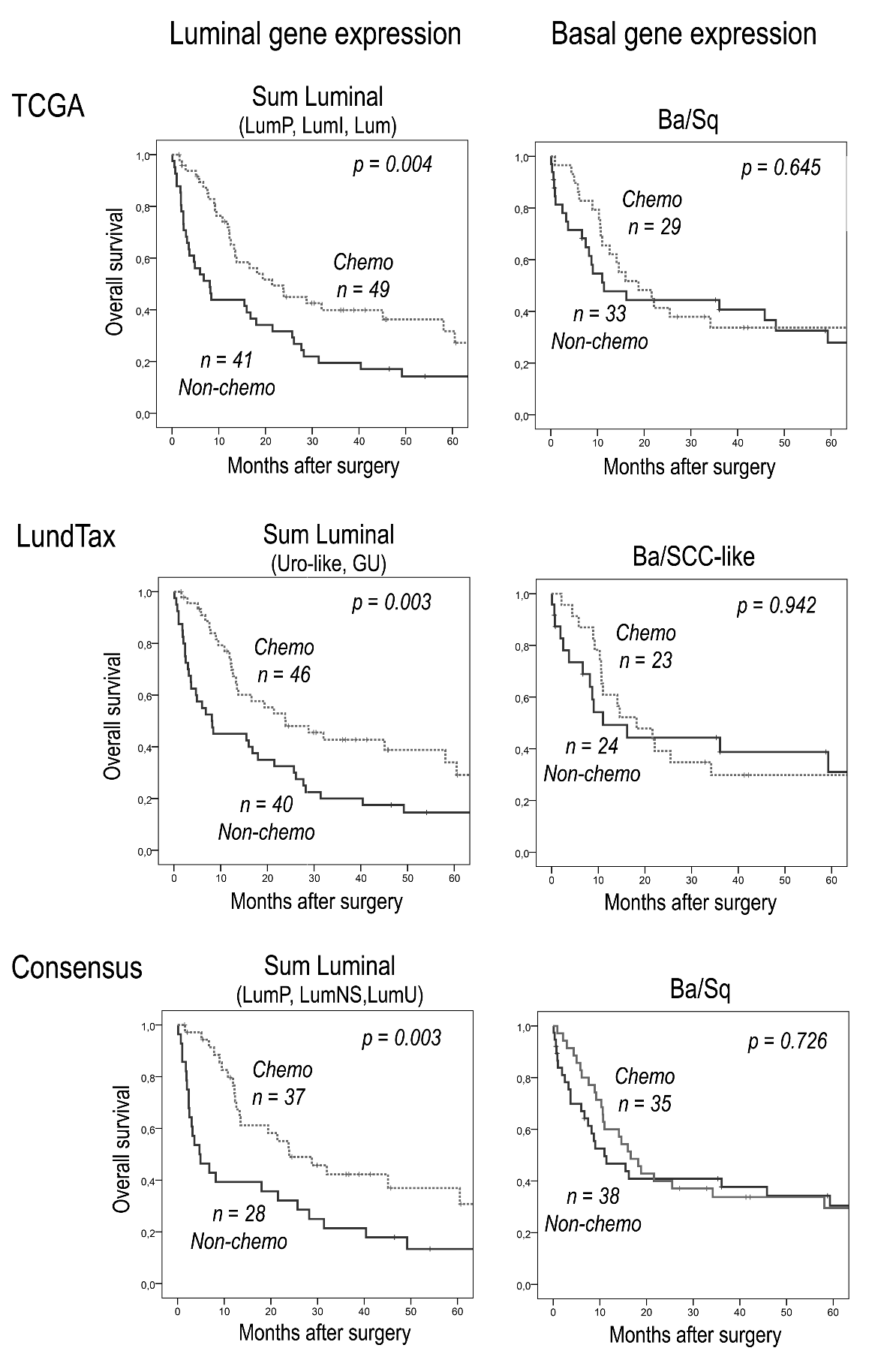


**Supplementary Figure 2.** Kaplan-Meier curves of overall survival stratified by merged sum Luminal molecular subtypes and basal subtypes. LumP: Luminal-papillary, LumI: Luminal-infiltrated, Lum: Luminal, Ba/Sq: Basal/Squamous, Uro-like: Urothelial-like, GU: Genomically unstable, Ba/SCC: Basal/SCC-like, LumNS: Luminal non specified, LumU: Luminal unstable.


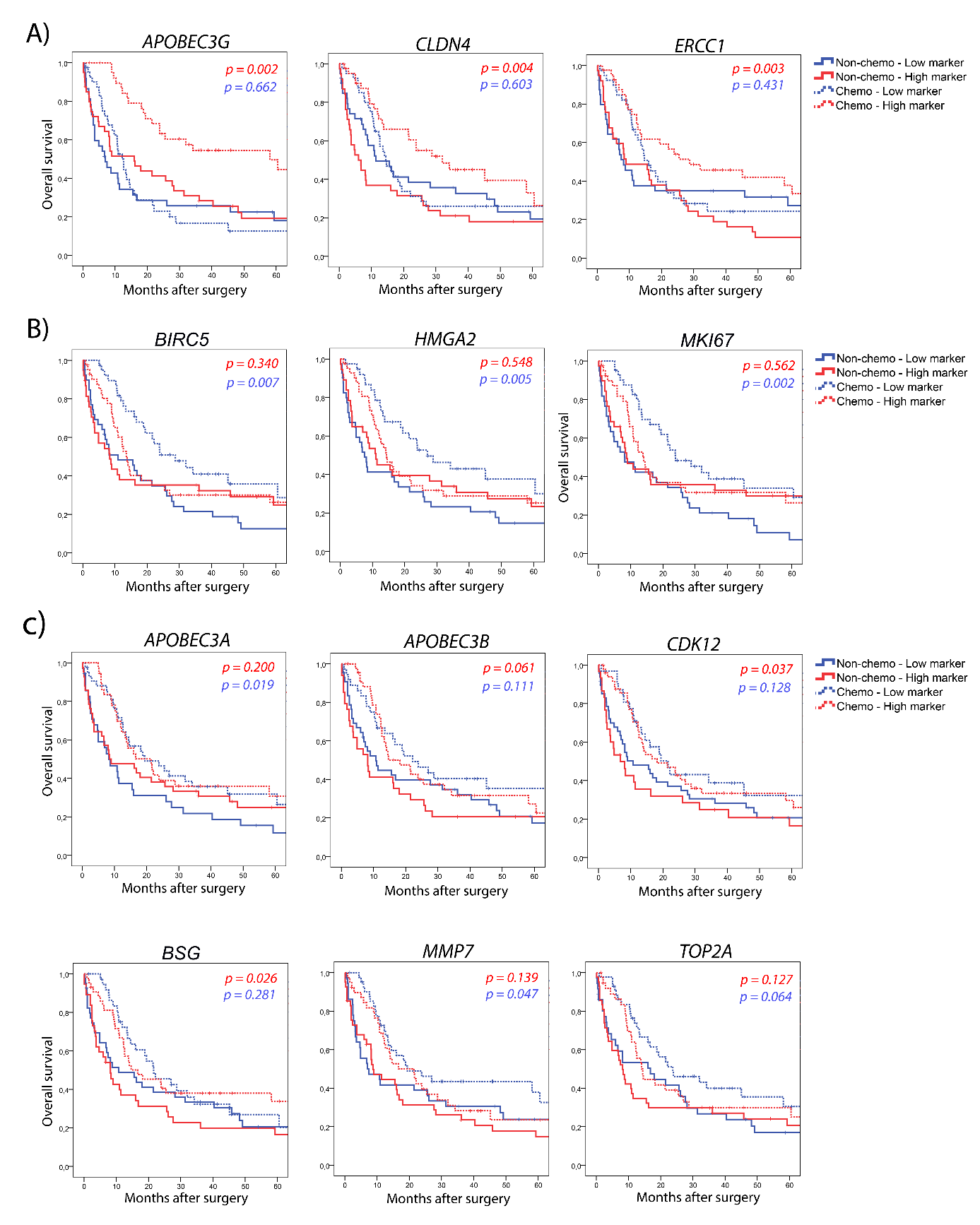


**Supplementary Figure 3.** Survival analyses stratified by gene expression levels of 12 single markers in platinum-treated (n=81) and untreated (n=78) patients. Patients with high *APOBEC3G*, *CLDN4*, and *ERCC1* (A) as well as those with low *BIRC5,* *HMGA2,* and *MKI67*, gene expression levels (B) derive significant overall survival benefit from platinum treatment. In contrast, *APOBEC3A, APOBEC3B, CDK12, BSG, MMP7,* and *TOP2A* (C) showed no difference in overall survival platinum-treated vs. untreated patients neither in the low nor in the high expression groups. P-values represent overall survival difference between platinum-treated and untreated patients in the subgroups with high gene expression (red) and low gene expression (blue) levels. P-values are provided separately for marker high (red) and marker low (blue) cases comparing chemo and non-chemo groups.


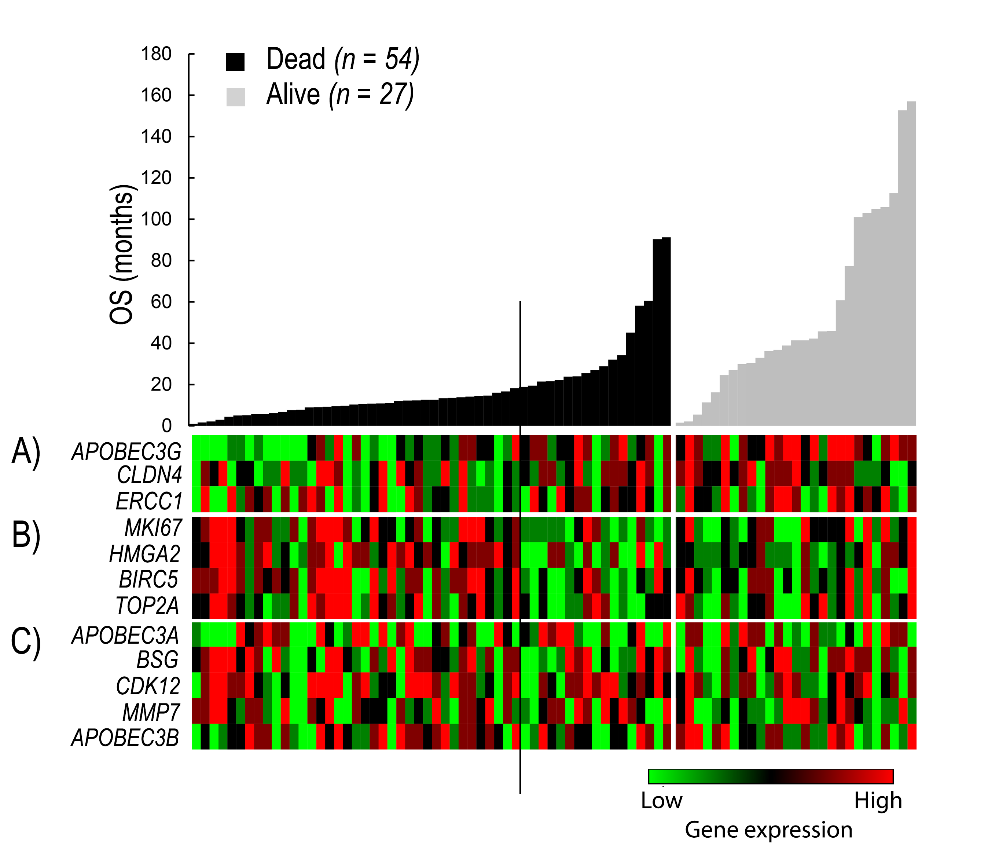


**Supplementary Figure 4.** Visual presentation of gene expression pattern of the selected 12 single markers in the chemotherapy-treated group, ordered according to OS time. Markers were divided into 3 groups: (A) genes, with high expression levels associated with longer survival (*APOBEC3G, CLDN4, ERCC1*), (B) genes, with high expressions correlated with worse OS (*MKI67, HMGA2, BIRC5, TOP2A*) and (C) genes, showing no correlation with OS (*APOBEC3A, APOBEC3B, BSG, CDK12, MMP7*). In marker groups (A) and (B), a switch in the gene expression pattern at ~19 months can be observed (vertical line). OS: overall survival.


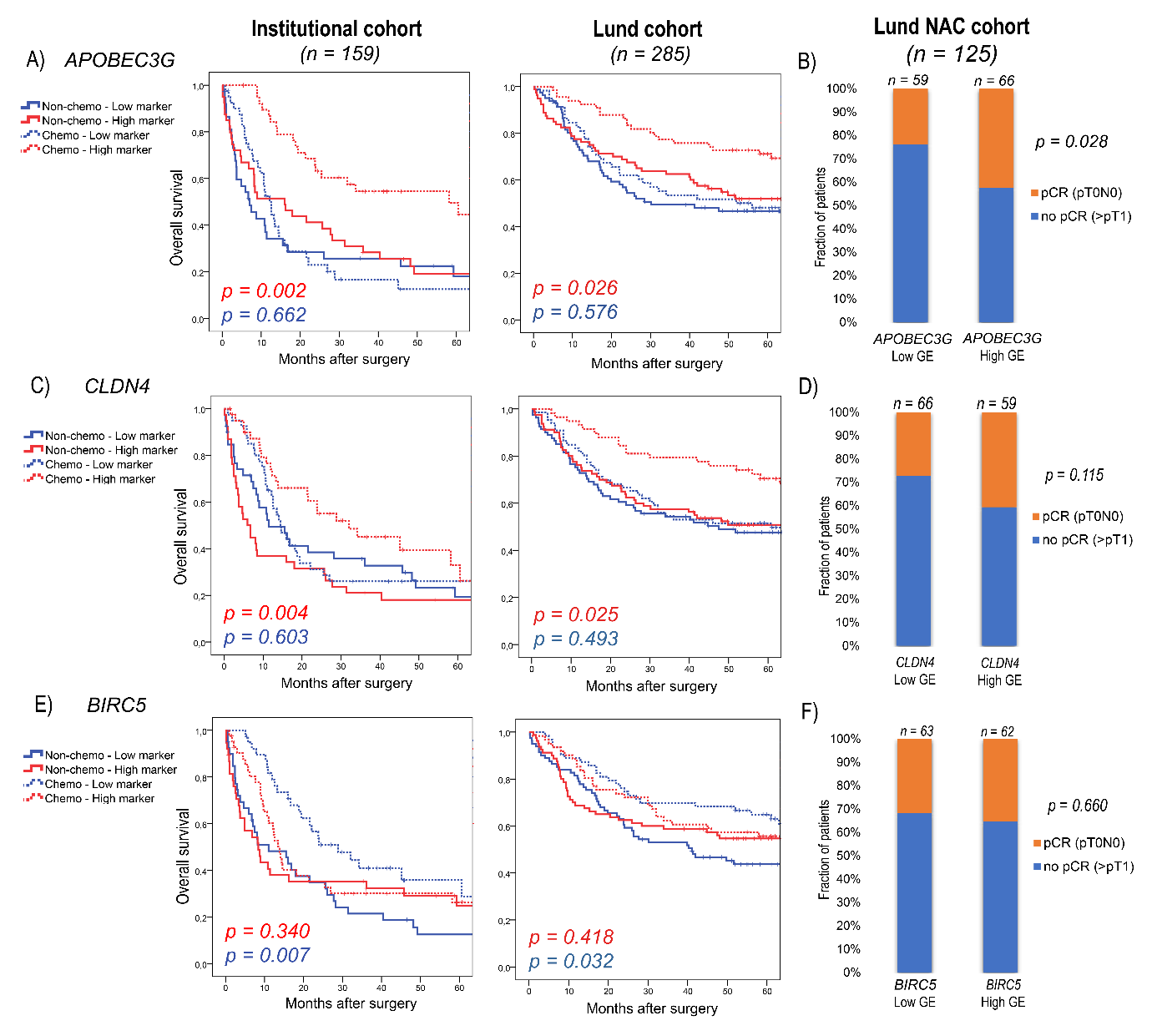


**Supplementary Figure 5.** Overall survival stratified by gene expressions of *APOBEC3G* (A), *CLDN4* (C) and *BIRC5* (E) in our institutional (n=159) and validation (Lund) (n=285) cohorts. Pathological response rate for neoadjuvant chemotherapy in the *APOBEC3G* (B), *CLDN4* (D) and *BIRC5* (E) low- and high gene expression groups in the Lund NAC cohort. P-values represent overall survival difference between platinum-treated and untreated patients in the subgroups with high (red) and low (blue) gene expression levels. pCR: pathological complete response rate.

*Supplementary Tables*

**Supplementary Table 1.** Markers and their applications in the present study. 48 subtype-specific markers for distinct molecular subtyping and the list of selected 12 potential chemo-predictive markers. “X” - marks signature-scores used for various subtype classification systems.

**Supplementary Table 2.** Correlation between molecular subtypes and clinicopathological parameters. LumP: Luminal-papillary, LumI: Luminal-infiltrated, Lum: Luminal, Ba/Sq: Basal/Squamous, Ne: Neuronal, Uro-like: Urothelial-like, GU: Genomically unstable, Mes-like: Mesenchymal-like, Ba/SCC-like: Basal/SCC-like, Sc/Ne-like: Small-cell/Neuroendocrine-like, LumNS: Luminal non specified, LumU: Luminal unstable, Ne-like: Neuroendocrine-like. P-values were calculated by using the Chi-square test; where one reference group was determined, and each subtype group was analyzed one-by-one. Age was applied as a continuous variable and the Mann-Whitney test was used for calculation differences between groups. Bold type represents statistically significant values (≤0.05).

**Supplementary Table 3.** Correlation between clinicopathological parameters and gene expression levels of 12 selected single markers. P-values with bold type are statistically significant (≤0.05).

**Supplementary Table 4.** Cox univariate (A) and multivariate (B; C) overall survival analyses. As signature scores were used for subtype classification; these cannot be included together with subtype classes in the same multivariate models. Therefore, two separate multivariate analyses were conducted; one included molecular subtypes (B); while the other the signature scores (C). HR: hazard ratio, CI: confidence interval, Ref: referent. LumP: Luminal-papillary, LumI: Luminal-infiltrated, Lum: Luminal, Ba/Sq: Basal/Squamous, Ne: Neuronal, Uro-like: Urothelial-like, GU: Genomically unstable, Mes-like: Mesenchymal-like, Sc/Ne-like: Small-cell/Neuroendocrine-like, LumNS: Luminal non specified, LumU: Luminal unstable, Ne-like: Neuroendocrine-like. CIS: in situ carcinoma, EMT: epithelial-to-mesenchymal transition, ECM: extracellular matrix. P-values with bold type are statistically significant (≤0.05).

**References**

1. Olah C, Hahnen C, Nagy N, Musial J, Varadi M, Nyiro G, Gyorffy B, Hadaschik B, Rawitzer J, Ting S, Sjödahl G, Hoffmann MJ, et al. A qPCR ‐based method for molecular subtype classification of urinary bladder cancer ‐ stromal gene expressions show higher prognostic values than intrinsic tumor genes. Int J Cancer 2021;150:856–67.
2. Robertson AG, Kim J, Al-ahmadie H, Weinstein JN, Kwiatkowski DJ, Lerner SP. Comprehensive Molecular Characterization of Muscle-Invasive Bladder Cancer. Cell 2017;171:540-556.e25.
3. Choi W, Porten S, Kim S, Willis D, Plimack ER, Hoffman-Censits J, Roth B, Cheng T, Tran M, Lee IL, Melquist J, Bondaruk J, et al. Identification of Distinct Basal and Luminal Subtypes of Muscle-Invasive Bladder Cancer with Different Sensitivities to Frontline Chemotherapy. Cancer Cell 2014;25:152–65.
4. Sjödahl G, Eriksson P, Liedberg F, Höglund M. Molecular classification of urothelial carcinoma: global mRNA classification versus tumour-cell phenotype classification. *J Pathol* 2017;242:113–25.
5. Kamoun A, de Reyniès A, Allory Y, Sjödahl G, Robertson AG, Seiler R, Hoadley KA, Groeneveld CS, Al-Ahmadie H, Choi W, Castro MAA, Fontugne J, et al. A Consensus Molecular Classification of Muscle-invasive Bladder Cancer. *Eur Urol* 2020;77:420–33.
6. Da Costa JB, Gibb EA, Bivalacqua TJ, Liu Y, Zarni Oo H, Miyamoto DT, Alshalalfa M, Davicioni E, Wright J, Dall’Era MA, Douglas J, Boormans JL, et al. Molecular characterization of neuroendocrinelike bladder cancer. *Clin Cancer Res* 2019;25:3908–20.
7. Grivas P, Bismar TA, Alva AS, Huang HC, Liu Y, Seiler R, Alimohamed N, Cheng L, Hyndman ME, Dabbas B, Black PC, Davicioni E, et al. Validation of a neuroendocrine-like classifier confirms poor outcomes in patients with bladder cancer treated with cisplatin-based neoadjuvant chemotherapy. *Urol Oncol Semin Orig Investig* 2019;38:262–8.
